# Sleep Deprivation Impairs Object-Selective Attention: A View from the Ventral Visual Cortex

**DOI:** 10.1371/journal.pone.0009087

**Published:** 2010-02-05

**Authors:** Julian Lim, Jiat Chow Tan, Sarayu Parimal, David F. Dinges, Michael W. L. Chee

**Affiliations:** 1 Neuroscience and Behavioral Disorders Program, Duke-NUS Graduate Medical School, Singapore, Singapore; 2 Division of Sleep and Chronobiology, Department of Psychiatry, University of Pennsylvania School of Medicine, Philadelphia, Pennsylvania, United States of America; University of Leuven, Belgium

## Abstract

**Background:**

Most prior studies on selective attention in the setting of total sleep deprivation (SD) have focused on behavior or activation within fronto-parietal cognitive control areas. Here, we evaluated the effects of SD on the top-down biasing of activation of ventral visual cortex and on functional connectivity between cognitive control and other brain regions.

**Methodology/Principal Findings:**

Twenty-three healthy young adult volunteers underwent fMRI after a normal night of sleep (RW) and after sleep deprivation in a counterbalanced manner while performing a selective attention task. During this task, pictures of houses or faces were randomly interleaved among scrambled images. Across different blocks, volunteers responded to house but not face pictures, face but not house pictures, or passively viewed pictures without responding. The appearance of task-relevant pictures was unpredictable in this paradigm. SD resulted in less accurate detection of target pictures without affecting the mean false alarm rate or response time. In addition to a reduction of fronto-parietal activation, attending to houses strongly modulated parahippocampal place area (PPA) activation during RW, but this attention-driven biasing of PPA activation was abolished following SD. Additionally, SD resulted in a significant decrement in functional connectivity between the PPA and two cognitive control areas, the left intraparietal sulcus and the left inferior frontal lobe.

**Conclusions/Significance:**

SD impairs selective attention as evidenced by reduced selectivity in PPA activation. Further, reduction in fronto-parietal and ventral visual task-related activation suggests that it also affects sustained attention. Reductions in functional connectivity may be an important additional imaging parameter to consider in characterizing the effects of sleep deprivation on cognition.

## Introduction

Although a broad array of cognitive processes are affected when human beings are deprived of sleep, deficits in sustained or vigilant attention are particularly robust and are of great importance in predicting real-world cognitive errors [1]. The decline in the capacity to maintain focus over extended periods has been well studied using behavioral and neuroimaging methods [2], [3]. In contrast, less is known about the effects of sleep deprivation (SD) on selective attention, which refers to the ability to focus cognitive resources on particular locations, objects, or features to the exclusion of irrelevant distracters. Existing studies on selective attention in the setting of sleep deprivation have yielded somewhat mixed results [4], [5], [6], [7], [8], [9]. One reason for this variability is that deficits in selective attention can accrue from a combination of sources [5], [10] which may not be dissociable using behavioral methods alone. In comparison, studying the neural substrates of attention using fMRI provides added dimensions along which to tease apart the contributions of specific deficits in selective attention from the dominant, non-specific effect of vigilance declines.

In the well-rested state, selective attention results in the biasing of sensory processing in favor of the attended stimulus over competing distracters [11]. This leads to topographically specific increases in neuronal firing rate [12], [13] and MR signal in sensory cortex [14]. Behavioral studies evaluating the effect of SD on selective attention suggest that despite an overall decline in response speed, feature-based visual search [5] and alerting may be relatively preserved [9].

Deficits in selective attention are likely to arise from a reduction in the strength of top-down biasing of information-processing in the sensory cortex. In support of this hypothesis, several functional neuroimaging experiments have shown that sleep deprivation in humans often results in reduced activation of the dorsal fronto-parietal attention network [8], [15], [16], [17], [18]. Crucially, however, these findings do not differentiate the effects of sleep deprivation on selective attention from other forms of attention as all forms generally recruit similar cognitive control areas. A useful alternative approach to identifying deficits in selective attention is to examine their downstream effects, for instance the influence of top-down biasing signals on activity in functionally differentiated and spatially dissociable sensory regions [19], [20].

In a recent experiment, subjects viewed picture quartets containing alternating faces and scenes with instructions to attend to faces, scenes, or both. In this paradigm, sleep deprivation reduced functional connectivity between the intraparietal sulcus (IPS) and the parahippocampal place area (PPA) [4]. However, while there was a main effect of state on PPA activation, modulation of PPA activity by attention was relatively preserved. Since the stimuli were presented in a regular and predictable order and timing, subjects could be thought of as being cued to respond to the target stimuli. Cues have been shown to ameliorate the effect of sleep deprivation on selective attention [9], [10], which may account for the preserved modulation of PPA in this prior study.

To investigate this hypothesis, we studied the effect of sleep deprivation on the functional anatomy of selective attention using a task that did not provide subjects with a prior alerting cue. We predicted that in addition to decreased activation in fronto-parietal control areas, we would also uncover reduced biasing of activation in the PPA to relevant stimuli. We additionally anticipated a reduction in connectivity between cognitive control regions and ventral visual cortex in the sleep-deprived as compared to the well-rested state.

## Materials and Methods

Twenty-seven undergraduates from the National University of Singapore were recruited for this within-subject study through advertisements on a campus website. From this original pool, two were removed from analysis due to excessive head-motion in the scanner, one was excluded based on near-chance performance in both states, and another was excluded on the basis of image problems, giving a final sample of N = 23 (12 male; mean age = 21.3 years, SD = 1.4 years). All subjects were right-handed, had no history of chronic physical or psychiatric disorders, or long-term medication use. They had regular sleep schedules and slept between 6.5–8 hours a night based on self-report, and were not extreme morning chronotypes as assessed by a modified Horne-Ostberg Chronotype Questionnaire [21].

Upon entering the study, subjects visited the lab for a briefing to practice the experimental task and to collect an Actiwatch (Actiwatch, Philips Respironics, USA) that they were instructed to wear at all times until the conclusion of the experiment. Subjects were also issued sleep diaries on which they were to record the onset and offset of all sleep bouts. Sleep history was checked prior to each of the fMRI scanning sessions, and participants who did not comply with a regular sleep schedule (>6.5 hours of sleep/night; sleep time no later than 1:00 AM; wake time no later than 9:00 AM) were excluded.

At least five days after the briefing, subjects returned to the laboratory for the first of two experimental sessions. In the rested wakefulness (RW) condition, subjects reported to the lab at approximately 7:30 AM. After filling in a questionnaire to assess their subjective level of sleepiness (the Karolinska Sleepiness Scale), they underwent an fMRI scan during which they performed a task involving selective attention to two different classes of stimuli: faces and houses (see fMRI procedures below for detailed description). Anatomical scans were also acquired during this time. fMRI scanning in the RW state typically began at about 8:00 AM. In the sleep deprivation (SD) condition, subjects reported to the lab on the evening prior to their fMRI scan. Subjects' actigraphy records were used to confirm they had awakened at their regular time on that day, and had not taken any daytime naps. Subjects remained awake overnight in the laboratory under the constant supervision of a research assistant. They were permitted to engage in light recreational activities, but were not allowed to smoke or consume caffeine. Every hour, participants performed the Psychomotor Vigilance Test and rated their subjective sleepiness using the Karolinska Sleepiness Scale. In the SD condition, subjects underwent an fMRI scan as in the RW condition, but at 6:00 AM. The order of scanning sessions was counterbalanced across subjects (RW session first; N = 12) to minimize potential order confounds. Sessions were separated by at least one week, so that subjects undergoing the SD session first had sufficient time to fully recover from the effects of sleep loss.

### Ethics Statement

**Figure 1:**
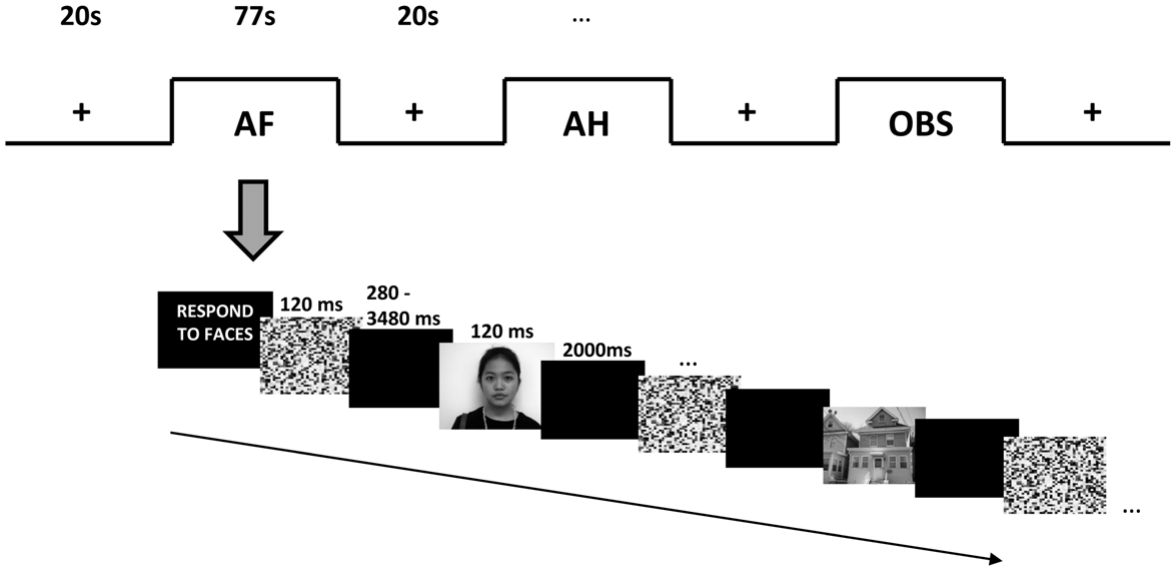
Schematic of the object-selective attention task. Three faces and three houses were presented during every task block. Inter-stimulus intervals varied randomly after each scrambled image, and were held constant at 2000 ms following each target. Subjects performed 6 task runs during each scanning session. AF  =  attend and respond to faces; AH  =  attend and respond to houses; OBS  =  passive observation of houses and faces.

Permission to conduct this study was granted by the Singapore General Hospital IRB, and all subjects provided written informed consent prior to participation. Subjects were financially compensated for their time. The individual providing the example face in Figure 1 provided written informed consent for the publication of this image.

### Experimental Paradigm

Subjects were shown blocks consisting of 6 novel targets (grayscale images of three faces and three houses) and 30 scrambled images that were of approximately equivalent luminance as the target pictures (Fig. 1). Equal numbers of male and female faces bearing neutral expressions were presented. Target stimuli were randomly interleaved with the scrambled images such that the interval between two targets ranged between 10 s and 14 s (mean = 12 s). The interstimulus interval for presentation varied randomly between 0.5 s and 3.5 s (mean = 1.75 s), except after the appearance of a target, when it was held constant at 2 s. This was to allow subjects adequate time to respond before the next stimulus onset.

At the start of each block, an instruction screen lasting 2 s was presented to the subject, informing them to either attend to faces, attend to houses, or passively observe the stimuli. This was followed by a further 2 s delay before the first stimulus appeared. In each of the ‘attend’ conditions, subjects were instructed to respond to the target by pressing a button with the right hand. In the ‘observe’ condition, subjects simply viewed the stimuli without making any response (Fig. 1). Thus, in the “attend to face” blocks, attend face (AF) and ignore house (IH) events were generated, and in “attend to house” blocks, attend house (AH) and ignore face (IF) events were generated. Observe face and observe house (OF and OH) events were generated in the blocks where stimuli were passively observed. fMRI runs consisted of 4 blocks of fixation (20 s) interleaved with 3 task blocks (77 s). Subjects performed 6 runs in total (all possible permutations of the task blocks) during each scanning session.

Finally, at the end of the RW session, subjects were scanned while they viewed blocks of faces and houses; data from these scans served as functional localizers that allowed us to identify the fusiform face area (FFA) and parahippocampal place area (PPA) for each individual subject [22]. Functional localizers consisted of eight stimulus blocks interleaved with nine fixation blocks, and lasted 6 minutes and 16 seconds each. Each stimulus block comprised either 18 faces or 18 houses, presented at the rate of 1 per second.

### Image Acquisition

MR imaging was conducted using a 3T Siemens Tim Trio scanner (Siemens, Erlangen, Germany) fitted with a 12-channel head coil. Participants viewed stimuli through a set of MR-compatible LCD goggles (Resonance Technology, Los Angeles, USA) and responded using their right index finger via a MR-compatible button box. Performance was continually monitored by a research assistant who noted all lapses and eye closures (through use of an eye tracking device). Subjects were prompted to attend to the task through an intercom system when they failed to respond to two consecutive trials, or when epochs of eye closure exceeded 3 seconds. Functional images were collected using a gradient echo-planar imaging sequence (TR: 2000 ms; TE: 30 ms; flip angle: 90°; field-of-view: 192×192 mm; matrix size: 64×64). Twenty-eight 3-mm axial slices aligned to the intercommisural plane and covering the whole brain were acquired. Directly following the functional data collection, a high-resolution T1 coplanar image was acquired. Finally, a high-resolution 3D MPRAGE sequence was obtained so that anatomical images could be normalized into common stereotactic space.

### Image Preprocessing and Analysis

MRI data were analyzed using Brain Voyager QX version 1.10.1 (Brain Innovation) and Matlab R13 (Mathworks). Functional images were aligned across scanning runs to the first image of the final run. Intrasession image alignment to correct for motion was performed using the first acquisition of the final functional run as the reference scan. Interslice timing differences within each functional acquisition were corrected using cubic spline interpolation. We performed Gaussian filtering in the spatial domain by applying an 8 mm FWHM smoothing kernel. Linear signal drift, and signals of lower than 3 cycles/functional run were removed. Finally, all images were registered to their respective individual 3D high-resolution T1 anatomical image, and normalized to Talairach space [23].

Functional imaging data were analyzed using a general linear model with 13 predictors in an event-related analysis. Twelve of these predictors were created with a 2×2×3 model using all combinations of state (RW/SD), stimulus type (house/face) and trial type (attend/observe/ignore). We modeled events by convolving a stick function with a double-gamma, canonical hemodynamic response. Only correct ‘attend’ responses were analyzed. A thirteenth predictor was created to model all lapses (non-responses within 2 s) in each state; these events were not subsequently analyzed any further. As we did not want to include periods of data that included frequent microsleeps, runs in which there were >50% of undetected targets were not entered into the model. We excluded 14 out of 288 runs (4.9%) from the analysis for this reason.

In order to identify cognitive control regions activated above threshold by selective attention to houses as well as faces, we computed the conjunction of two contrasts: attend house (AH) vs. baseline and attend face (AF) vs. baseline in the RW state. To control for Type I error, voxels were processed using an iterative cluster size thresholding procedure [24] that considered the spatial smoothness of functional imaging data when generating activation maps based on a corrected cluster threshold (*p*<.05). Subsequent to this, a voxel-level threshold of at least *p*<.001 (uncorrected) for *t* maps was applied.

To characterize state-related differences in control region activation during task performance, we compared activation within a 10×10×10 mm cube of voxels surrounding the peak voxels obtained from the conjunction analysis described above in addition to running an ANOVA-based analysis. The frontal and parietal regions selected from the conjunction analysis have previously been identified as important areas involved in selective attention [4], [25]. These ROIs were then interrogated to evaluate the relative magnitude of activation for attend, ignore and observe conditions across the two states. All secondary statistical tests were conducted using SPSS version 17.0 (SPSS Inc., Chicago, IL).

Analysis of object-selective attention within the ventral visual cortex was ROI-based. The PPA and FFA were defined by a separately conducted localizer scan performed for each individual as described previously. PPA ROIs comprised a 10×10×10 mm cube of voxels that surrounded the one voxel showing maximum difference in activation between house and face blocks. We focused our analysis on the PPA as it has been shown to yield more discriminating and spatially more consistent, selectivity data [4], [19], [20]. Furthermore, because there was no hemispheric asymmetry of PPA activation, activation magnitude data for all conditions—AH (attend house), IH (ignore house) and OH (observe house)—were obtained from both the left and right PPA and averaged. Activation magnitude across trial type and state was evaluated using paired t-tests. We opted not to use analysis of variance (ANOVA) as we had specific a priori hypotheses, and because some of the comparisons in the 2-way ANOVA would not have been meaningful (e.g. AH_RW_ vs OH_SD_).

Psychophysiological interaction (PPI) analysis [26] was performed by extracting the time series of activation from a 10 mm cubic region around the peak voxels identified by the conjunction of AH vs. baseline and AF vs. baseline contrasts within the left intraparietal sulcus (IPS; Talairach co-ordinates: −27, −58, 37) as well as the left inferior frontal gyrus/insula (Talairach co-ordinates: −36, 11, 4). We selected these regions due to their known involvement in biasing object-based attention, and for consistency with a companion study [4].

To carry out PPI analysis, we used a linear model with three predictors: the time course of activity in the seed ROI, a task predictor coding for activity within task blocks (AH vs. IH or AH vs. OH) and a PPI term. To construct the PPI term, the deconvolved time-course of the relevant seed region was multiplied with a vector containing the psychological variables of interest. This product was then re-convolved with a canonical hemodynamic response function [27]. The coefficient of this third, interaction term, is the one of interest in PPI analyses. Statistical maps of functional connectivity for each state were computed by conducting two-tailed, one sample t-tests on parameter estimates of the PPI (RW and SD) thresholded at *p*<.05.

To evaluate the robustness of the findings, we compared PPI in the AH vs. IH as well as AH vs. OH contexts as both comparisons evaluate object-selective attention.

## Results

### Behavioral Data

In the RW state, subjects were able to perform the task accurately with high hit rates (mean = 91.0%, SD = 11.0%) and low rates of false alarms (mean = 4.1%, SD = 4.6%). After sleep deprivation, there was a significant decline in the percentage of hits (*t*_22_ = 5.30, *p*<.001); however, there was no significant change in the percentage of false alarms, and reaction times were not significantly different across state (Table 1). There were no significant differences in performance accuracy observed between face and house detection blocks in either state.

**Table 1:** Behavioral data from the selective attention task (N = 23).

Behavioral variable	RW	SD	t value
Hits (%)	91.05 (10.98)	75.48 (17.13)	5.30*
False alarms (%)	4.11 (4.57)	4.95 (5.09)	−0.63
Mean reaction time (ms)	574.08 (82.97)	593.48 (83.99)	−1.24
Subjective sleepiness	4.65 (1.78)	8.40 (0.71)	−10.1*

Data were collapsed across Attend House (AH) and Attend Face (AF) blocks. Subjective sleepiness was measured using the Karolinska Sleepiness Scale.

*p<.001.

### Brain Activation Associated with Selective Attention

**Figure 2:**
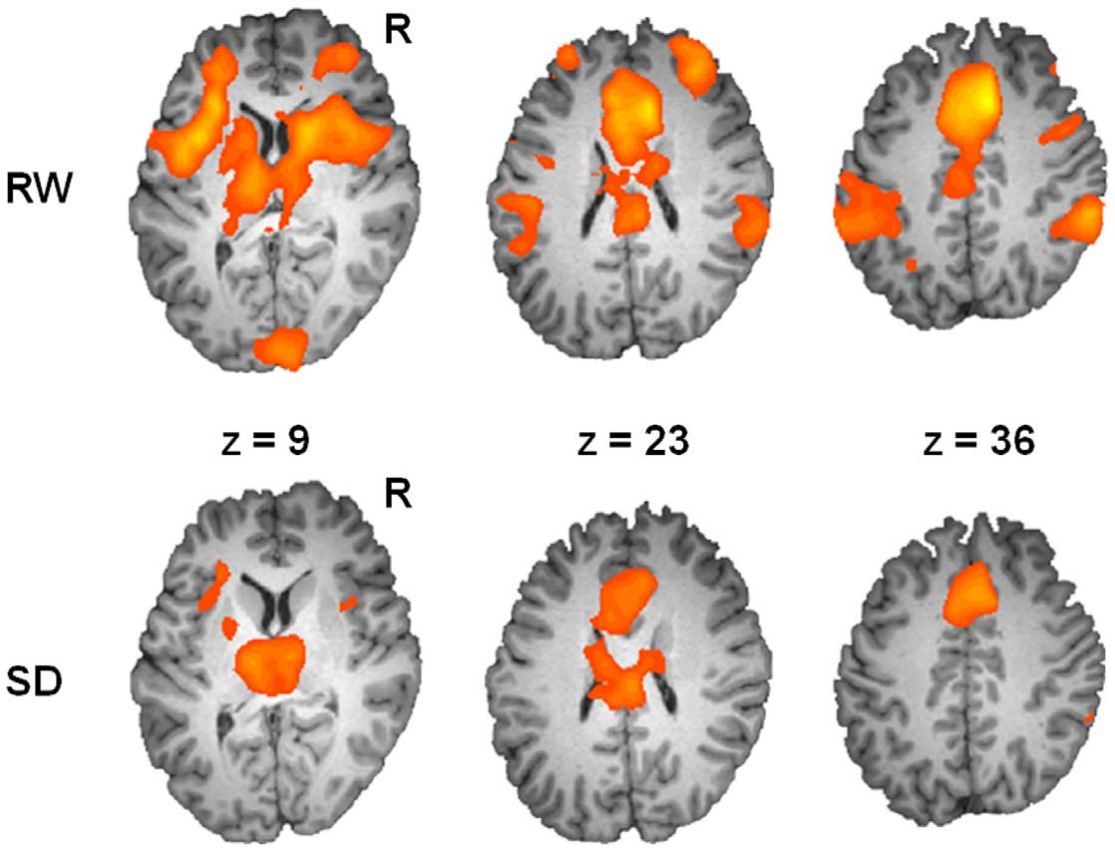
Effect of selective attention task on brain activation. Brain regions showing significant activation in the conjunction of Attend House (AH) vs. baseline and Attend Face (AF) vs. baseline conditions (*p*<.001, uncorrected). The top panel depicts activation during rested wakefulness (RW), and the bottom panel depicts activation after approximately 24 h of total sleep deprivation (SD).

Brain regions activated as a result of attending to houses as well as faces included the intraparietal sulcus (IPS) and inferior parietal lobes bilaterally (BA 40), left inferior frontal gyrus, right middle frontal gyrus, anterior cingulate cortex (Table 2), the thalamus, anterior areas of the frontal lobe (Fig. 2) as well as the ventral visual cortex.

**Table 2:** Talairach coordinates of activation peaks in regions potentially mediating cognitive control identified by the conjunction of Attend House (AH) vs. baseline and Attend Face vs. baseline trials (*p*<.001 uncorrected).

Region	BA	Talairach coordinates			*t* value	
		x	y	z	RW	*SD*
L intraparietal sulcus	7/40	−27	−58	37	4.48**	1.06
R intraparietal sulcus	7/40	33	−58	43	4.69***	2.69*
L superior frontal gyrus	10	−24	47	5	3.10**	1.21
R superior frontal gyrus	10	30	50	22	4.65***	3.73**
R middle frontal gyrus	46	24	44	−5	3.74***	2.61*
L inferior frontal gyrus	13	−36	11	4	4.97***	4.01**
Anterior cingulate cortex	32	−9	11	43	5.37***	4.57***

BA  =  Brodmann's area.

*p<.05.

**p<.01.

***p<.001.

**Figure 3:**
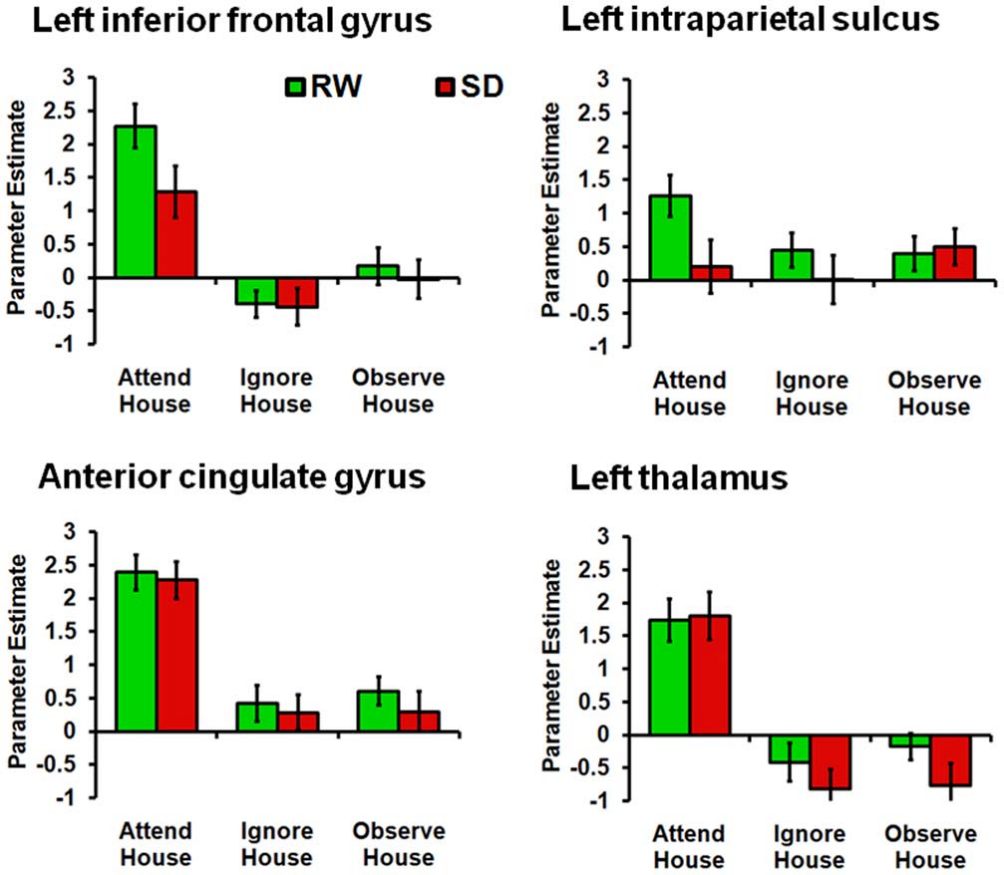
Parameter estimates of activation for the house conditions in areas associated with arousal and attention. Parameter estimates for each condition and state associated with the left inferior frontal gyrus (IFG), left intraparietal sulcus (IPS), anterior cingulate cortex (ACC), and left thalamus. Significant state-related differences were observed in the left IFG and IPS, but not in ACC or the thalamus.

Attending to houses elicited greater activation than ignoring houses in the left IPS (*t*_22_ = 2.72, *p*<.05), left inferior frontal regions (*t*_22_ = 6.83, *p*<.001), anterior cingulate cortex and the thalamus (ACC: *t*_22_ = 7.61, *p*<.001; thalamus: *t*_22_ = 6.47, *p*<.001; Fig. 3). Similar modulation of attention in the three cognitive control regions as well as the thalamus was observed when attending to faces as opposed to ignoring or observing them (Fig. S1). In subsequent analyses, we focused on the effect of attending to houses because of the clearer effects of attention on PPA activation as described in previous studies [4], [19], [20].

After a normal night of sleep (RW), attending to houses resulted in greater activation in the PPA in both contrasts of interest AH vs. IH (*t*_22_ = 2.36, *p*<.05) and AH vs. OH (*t*_22_ = 3.14, *p*<.01). After correcting for the two comparisons, the former contrast dropped just below the level of statistical significance (*p* = .056). Nevertheless, effect sizes for these comparisons were in the moderate to large range (*d* = 0.57 and 0.68 respectively). To verify that this effect was not spurious, we repeated the analysis using the PPA peak in the group map for reference instead of an individually selected PPA ROI. This resulted in finding significant AH vs. IH (*t* = 2.99, *p* = .006) and AH vs. OH (*t* = 3.25, *p* = .004) contrasts in RW, which would have survived Bonferroni correction. AH vs. IH and AH vs. OH comparisons in SD around this voxel failed to reach statistical significance (*t* = 0.25, *p* = .81 and *t* = 1.36, *p* = .19 respectively).

### Effects of Sleep Deprivation on Activation

**Figure 4:**
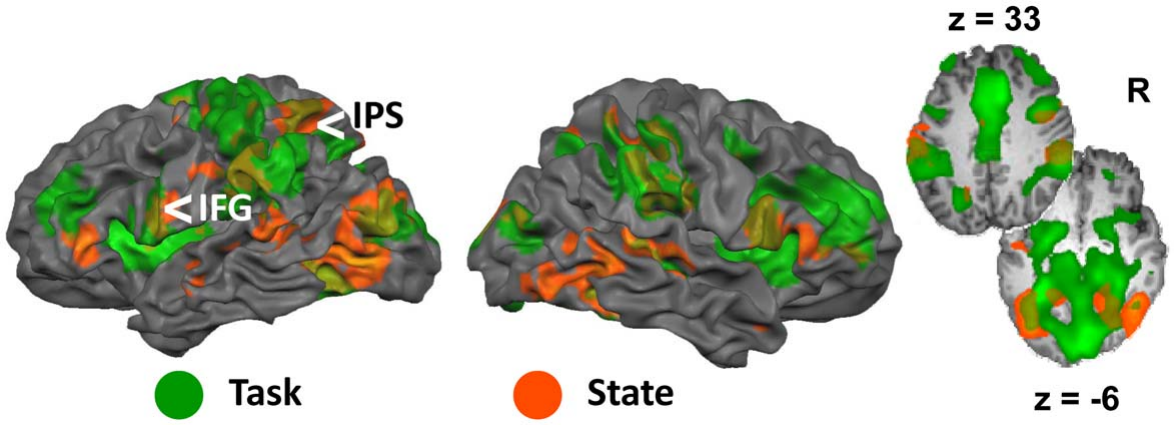
Effect of sleep deprivation on activation associated with selective attention for houses. Brain regions that showed a significant effect of state on activation in the Attend House (AH) vs. baseline contrast (*p*<.001 uncorrected; in orange). This finding was similar to the main effect of state obtained using an ANOVA analysis. For comparison, the regions showing the effect of task are overlaid in green and the overlap between regions showing task and state effects are in an intermediate color. IPS  =  intraparietal sulcus; IFG  =  inferior frontal gyrus.

**Figure 5:**
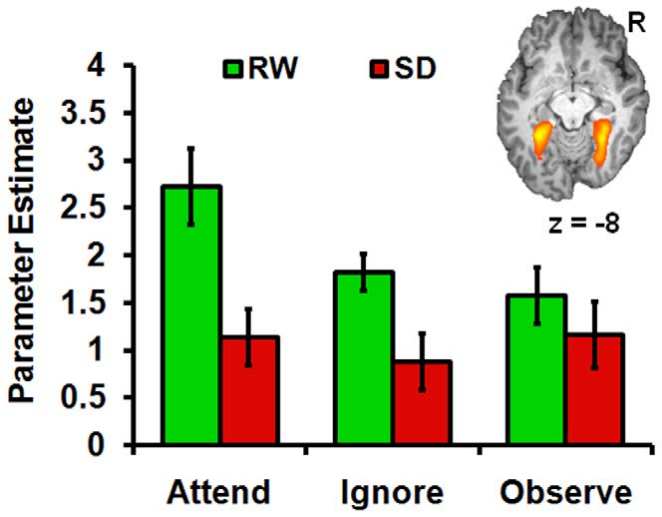
Effects of sleep deprivation and attention on parahippocampal place area (PPA) activation. In the rested (RW) state, attention to houses (AH) resulted in significantly greater PPA activation compared to ignoring (IH) or observing (OH) houses. However, this attention biasing was lost during SD.

SD reduced activation in the left inferior frontal ROI (*t*_22_ = 2.50, *p*<.05) and left IPS (*t*_22_ = 2.41, *p*<.05; Figs. 3 and 4) in the attend conditions but did not affect activation in the anterior cingulate (*t*_22_ = 0.41, *n.s.*) or the thalamus (*t*_22_ = 0.23, *n.s.*). These regions also appeared when probing for a main effect of state using an ANOVA approach (Fig. 4). The biasing effect of attention on PPA activation evident during RW was significantly attenuated following SD (Fig. 5). Paired t-tests between AH vs. IH and AH vs. OH in the SD condition were not significant at the *p*<.05 level (effect sizes: *d* = 0.18 and −0.01 respectively). Moreover, there was a significant effect of state when comparing activation in the AH condition relative to baseline (*t*_22_ = 3.93, *p*<.001).

### Psychophysiological Interaction (PPI) Analysis

**Figure 6:**
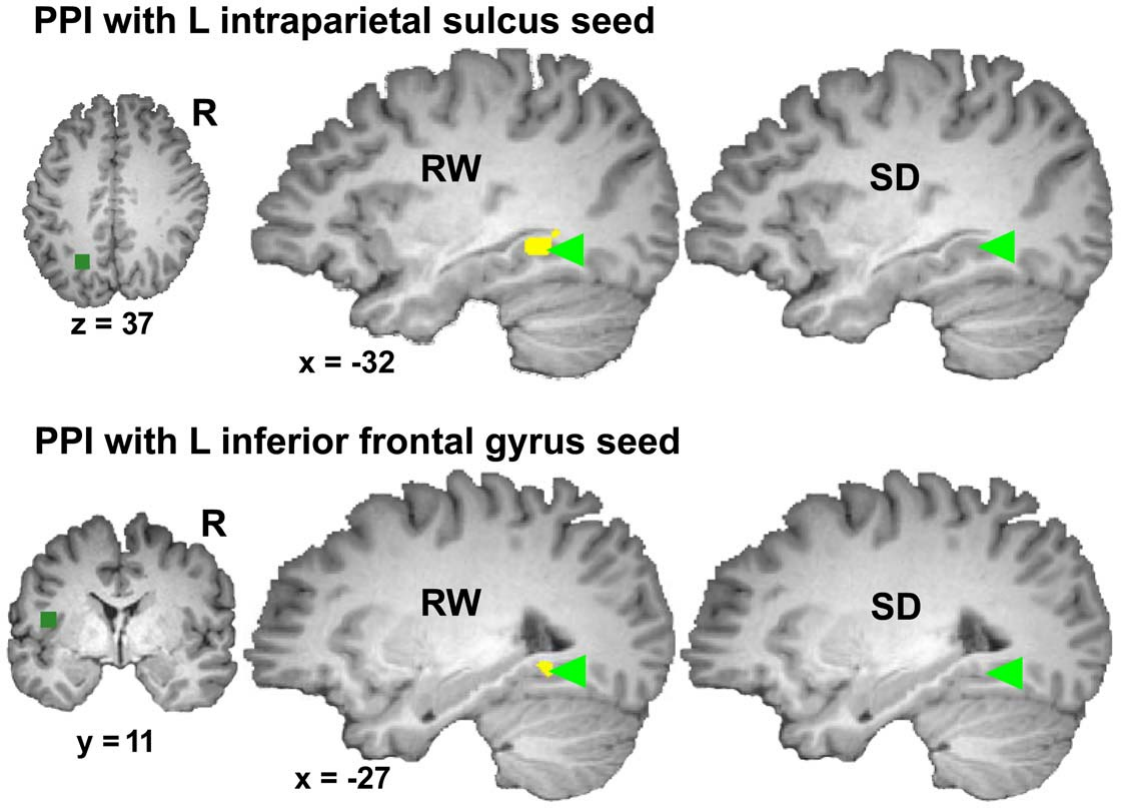
Psychophysiological interaction (PPI) in the rested and sleep deprived state. Connectivity analysis was performed using seeds in the left intraparietal sulcus (IPS; Talairach co-ordinates: −27, −58, 37) and left inferior frontal regions (Talairach co-ordinates: −36, 11, 4) (seed regions represented by green squares). Each map represents the conjunction of regions showing significant PPI in the Attend House (AH) vs. Ignore House (IH) and AH vs. Observe House (OH) conditions (threshold *p*<.05).

Whole-brain PPI analysis revealed significant connectivity between the seed voxels in the left IPS and the PPA bilaterally during RW (AH vs. IH: *t*_22_ = 4.77, *p*<.001; AH vs OH: *t*_22_ = 3.34, *p*<.01) but not following SD (AH vs. IH: *t*_22_ = 1.52, *n.s.*; AH vs OH: *t*_22_ = 1.31, *n.s.*; Table 3, Fig. 6, Fig. S2). Using a paired t-test, the direct comparison of PPI values across state for the PPA was significant only for AH vs. IH (AH vs. IH: *t*_22_ = 1.88, *p*<.05, 1-tailed; AH vs. OH: *t*_22_ = 0.73, *n.s.*). A separate PPI analysis evaluating connectivity between the left inferior frontal gyrus/insula and other brain areas found significant interaction between the left frontal seed and the PPA following a night of normal sleep (AH vs. IH: *t*_22_ = 2.67, *p*<.05; AH vs OH: *t*_22_ = 3.31, *p*<.01) but not following SD (AH vs. IH: *t*_22_ = 1.05, *n.s.*; AH vs OH: *t*_22_ = 0.48, *n.s.*; Table 3, Fig. 6, Fig. S2). Comparing the PPI across state for the PPA, we found a significant difference in the AH vs. IH comparison (AH vs. IH: *t*_22_ = 2.69, *p*<.05; AH vs. OH: *t*_22_ = 1.27, *n.s.*).

**Table 3:** Parietal and frontal seed regions showing psychophysiological interaction with the PPA (Talairach co-ordinates shown) under different task conditions.

Seed region	Contrast	Talairach coordinates of PPA region showing PPI			*t* value	
		x	y	z	RW	SD
L parietal (−27,−58,37)	AH > IH	−33	−44	−8	4.77***	1.52
	AH > OH	−35	−41	−4	3.34***	1.31
L inferior frontal gyrus (−36,11,4)	AH > IH	−27	−48	−8	2.67*	1.05
	AH > OH	−24	−46	−6	3.31**	0.48

Seeds for this analysis were in left parietal and left inferior frontal regions. *t* values denote the significance of the PPI term determined separately for each state.

*p<.05.

**p<.01.

***p<.001.

## Discussion

Three key findings were of interest in the present study. First, we found that sleep deprivation attenuated connectivity between the IPS and the PPA when selective attention for houses was engaged, replicating our previous report [4]. Secondly, SD eliminated the biasing effect of attention on PPA activation. Finally, the reduction in fronto-parietal and PPA activation in the sleep deprived state supports the notion that performance decline in the selective attention task may be caused by both specific deficits in selective attention as well as non-specific changes in sustained attention as reported in previous imaging studies [4], [28]

Although inter-individual differences in vulnerability to sleep deprivation [29], [30] can partially explain the differences in behavioral performance reported in various studies, another factor to consider is the extent to which the cognitive function of interest is actually affected by SD. Speed and accuracy of performance are almost always modulated by several subcomponents within a given cognitive task [31]. For example, when evaluating visual search in the setting of sleep deprivation, it was found that search speed did not decrease with increasing search set size [5]. Instead, SD-related response slowing was uniform across search set size suggesting that a non-search-related factor was responsible for performance decline. Along similar lines, an experiment intended to study visual short term memory revealed imaging changes that implied a deficit in attention and/or visual processing rather than in memory capacity [15]. Finally, a meta-analysis of behavioral changes induced by sleep deprivation indicated that the effect sizes associated with decrements in non-specific processes such as vigilance or sustained attention are relatively large [32] when compared to other more complex tasks.

Although imaging studies can shed light on functional neuroanatomy, studies that focus their analysis on top-down control regions, which include prefrontal and parietal areas, typically do not decompose total activation into the relative contributions of component cognitive processes [33]. However, by assaying activation in spatially differentiated regions in the ventral visual pathway that are the targets of object-selective attention [19], [20], [34], we were able to determine how object-selective attention was affected by sleep deprivation.

### Sleep Deprivation Reduces Connectivity between the Parietal/Frontal and Ventral Visual Areas

In a related study [4], it was suggested that functional connectivity might be a useful technique to detect deficits in object-selective attention. The current results use an event-related design to provide converging evidence for this claim.

In order to reveal a state-related change in PPI, MR signal in the ‘target’ area has to show consistent trial-by-trial differences in co-variation of signal with that of the seed region involving both task and non-task related aspects of the signal. This represents a different aspect of how attention might modulate BOLD signal (as opposed to the more intuitive demonstration of selectivity in PPA activation as a function of attention).

### Sleep Deprivation Affects Attention-Biased Changes in PPA Activation in the Absence of a Stimulus Cue

In contrast to the related study [4], subjects in the current experiment were unable to predict whether they would encounter a house or a face picture. We posit that this may explain why SD interacted with attention to modulate PPA activation in the present work.

The presence of a valid cue significantly reduces response time in experiments evaluating spatial attention [35]. In sleep-deprived persons, availability of a neutral or valid cue has been shown to afford preserved performance whereas invalid cues result in delayed responses. It has been postulated that the alerting (warning) effect of a cue, as opposed to re-orienting, is relatively preserved in sleep-deprived persons [9].

Orienting recruits the parietal lobe [36] and patients with parietal lobe lesions show deficits in performance during invalid and uncued trials [37]. Coincidentally, reduced task-related activation of the dorsal parietal region is a frequent finding in sleep-deprived persons [8], [15], [17], [18], [38]. In contrast, alerting recruits the thalamus [36] whose activation is often relatively preserved in multiple experiments evaluating attention following SD [4], [8], [28].

The availability of a valid cue may benefit behavior [9], [10]. When a cue is not available, as in the case of the present experiment, selective attention may deteriorate during SD, accompanied by a corresponding failure in the modulation of PPA activation. We acknowledge that the framework we have appealed to was originally used to explain behavior in the context of spatial attention [35]. However, the parsimony of the present and prior findings indicates that the framework may also apply to object-based attention.

### Changes Across State in Task-Related Activation

In addition to the changes in PPI and in PPA activity modulation, sleep deprivation also resulted in significant reductions in activation across conditions in inferior frontal regions, IPS and ventral visual cortex. These state-related changes in activation are consistent with prior studies from our laboratory on visual short-term memory [15], [38], working memory [17] and lapses of attention [18]. These changes in activation are thought to relate to a loss of sustained attention or a general visual processing resource that cuts across multiple tasks.

We posit that in experiments where sustained attention is a major contributor to the behavioral effect, state-related changes in activation will correlate with the corresponding change in behavior [17], [38]. On the other hand, activation-behavior correlations may not be found for tasks in which both sustained and selective attention contribute variance to the final outcome, as in the case of our two selective attention studies [4].

### Conclusion

Using a novel imaging paradigm and an analysis strategy that focused on the ventral visual cortex, we were able to dissociate the brain activation changes that reflect how sleep deprivation influences selective attention from task-independent changes in brain activation that involve cognitive control and higher visual areas. For selective attention tasks, reductions in connectivity between cognitive control regions and relevant visual areas appear to be a consistent feature of neural activity following SD. Finally, the absence of a cue in the present paradigm could explain the loss of the biasing effect of attention on PPA activation in sleep-deprived persons.

## Supporting Information

Figure S1 Parameter estimates of activation for faces in areas associated with arousal and attention. Parameter estimates for each condition and state in the left inferior frontal gyrus (IFG), left intraparietal sulcus (IPS), left thalamus and anterior cingulate cortex (ACC) for the three conditions attend to face, ignore face, and observe face. Significant state-related differences were observed in the left IFG and IPS, but not in ACC or the thalamus, mirroring the results for the house conditions in Fig. 4
(0.18 MB TIF)

Figure S2 Psychophysiological interaction related to the specific PPI contrasts and state. Connectivity analysis was performed using seeds in the left IPS (top panel; Talairach co-ordinates: −27, −58, 37) and left inferior frontal regions (bottom panel: Talairach co-ordinates: −36, 11, 4). Each map represents regions showing significant PPI in the AH vs. IH and AH vs. OH conditions (threshold p<.05) and in each state (RW, SD).
(0.42 MB TIF)
