# Supplementary figures and images for: Sleep Deprivation Impairs Object-Selective Attention: A View from the Ventral Visual Cortex
Source: PLoS One. 2010 Feb 5;5(2):e9087. doi: 10.1371/journal.pone.0009087 (PMC2816724; doi:10.1371/journal.pone.0009087)

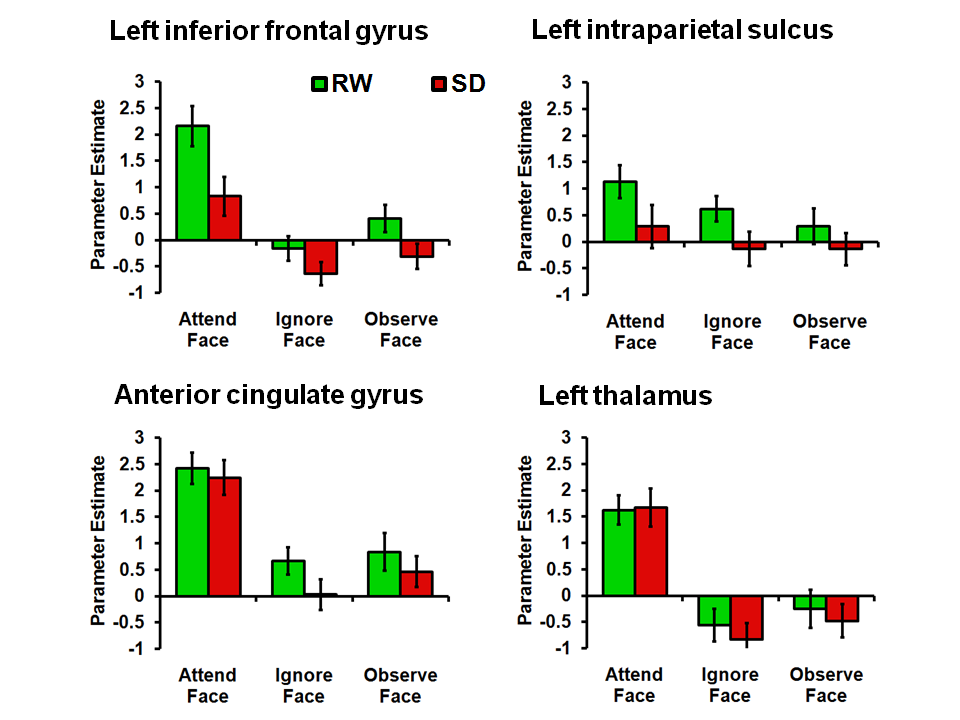

Supplement: Figure S1 — Parameter estimates of activation for faces in areas associated with arousal and attention. Parameter estimates for each condition and state in the left inferior frontal gyrus (IFG), left intraparietal sulcus (IPS), left thalamus and anterior cingulate cortex (ACC) for the three conditions attend to face, ignore face, and observe face. Significant state-related differences were observed in the left IFG and IPS, but not in ACC or the thalamus, mirroring the results for the house conditions in Fig. 4 (0.18 MB TIF) [file pone.0009087.s001.tif]

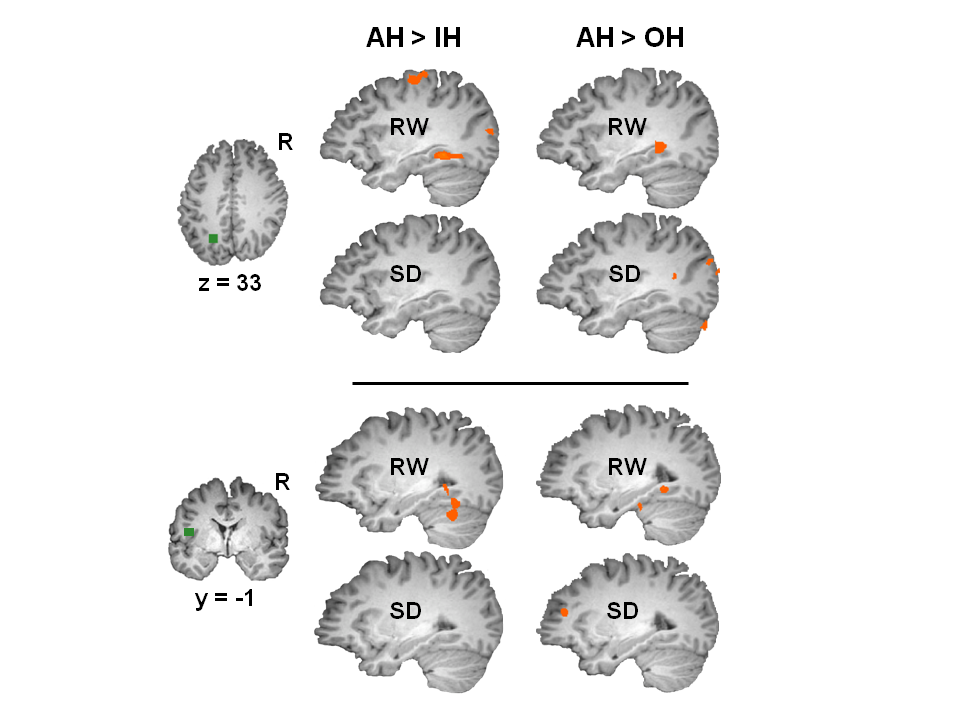

Supplement: Figure S2 — Psychophysiological interaction related to the specific PPI contrasts and state. Connectivity analysis was performed using seeds in the left IPS (top panel; Talairach co-ordinates: −27, −58, 37) and left inferior frontal regions (bottom panel: Talairach co-ordinates: −36, 11, 4). Each map represents regions showing significant PPI in the AH vs. IH and AH vs. OH conditions (threshold p<.05) and in each state (RW, SD). (0.42 MB TIF) [file pone.0009087.s002.tif]
